# Experience and Satisfaction with a Family-Based Physical Activity Intervention Using Activity Trackers and Apps: A Qualitative Study

**DOI:** 10.3390/ijerph20043327

**Published:** 2023-02-14

**Authors:** Stephanie Schoeppe, Kim Waters, Jo Salmon, Susan L. Williams, Deborah Power, Stephanie Alley, Amanda L. Rebar, Melanie Hayman, Mitch J. Duncan, Corneel Vandelanotte

**Affiliations:** 1 Physical Activity Research Group, Appleton Institute, School of Health, Medical and Applied Sciences, Central Queensland University, Rockhampton 4702, Australia; 2 Institute for Physical Activity and Nutrition (IPAN), School of Exercise and Nutrition Sciences, Deakin University, Geelong 3125, Australia; 3 Priority Research Centre for Physical Activity and Nutrition, School of Medicine and Public Health, Faculty of Health and Medicine, The University of Newcastle, Newcastle 2308, Australia

**Keywords:** family, program, children, parents, motivation, physical activity, smartphone, tablet, fitness trackers, wearables

## Abstract

Wearable activity trackers and smartphone apps have been shown to increase physical activity in children and adults. However, interventions using activity trackers and apps have rarely been tested in whole families. This study examined the experience and satisfaction with an activity tracker and app intervention (Step it Up Family) to increase physical activity in whole families. Telephone interviews were conducted with Queensland-based families (n = 19) who participated in the Step it Up Family intervention (N = 40, single-arm, pre/post feasibility study) in 2017/2018. Using commercial activity trackers combined with apps, the intervention included an introductory session, individual and family-level goal setting, self-monitoring, family step challenges, and weekly motivational text messages. Qualitative content analysis was conducted to identify themes, categories and sub-categories. In summary, parents reported that children were engaged with the activity tracker and app features to reach their daily step goals. Some technical difficulties were experienced with app navigation, syncing of activity tracker data, and tracker band discomfort. Although families liked that the weekly text messages reminded them to be active, they did not find them very motivating. Using text messages for physical activity motivation in families requires further testing. Overall, the intervention was well-received by families for increasing physical activity motivation.

## 1. Introduction

The Australian physical activity guidelines recommend at least 60 min of moderate-to-vigorous physical activity (MVPA) per day for children aged 5–17 years and at least 150 min of MVPA per week for adults aged 18 years and over [1,2]. In Australia, only 19% of children aged 5–17 years and 45% of adults meet these national guidelines [3,4]. Physical inactivity tends to start in early childhood, exacerbates during adolescence, and continues throughout adulthood [3,5]. Insufficient physical activity is detrimental to the health of children and adults. In children, physical inactivity contributes to reduced cardiorespiratory fitness, insufficient motor skills, excess weight, and the metabolic syndrome [6,7,8]. In adults, physical inactivity contributes to the development of major chronic diseases, such as cancer, coronary heart disease, and type 2 diabetes [9]. As such, physical inactivity is an important public health issue, and it costs the Australian health care system $13.8 billion annually in healthcare costs [10].

Physical inactivity often runs in the family [11]. The family is a unit where children and parents influence each other’s physical activity behaviours through mutual support and role modelling [11,12,13,14,15,16]. Therefore, family-based physical activity interventions need to actively involve the whole family [17,18]. However, previous physical activity interventions in families have involved parents mainly as supporters of their child’s physical activity engagement but less so as actively involved participants [18,19]. As such, few interventions in this area have involved the whole family by promoting physical activity participation in both children and parents [20,21,22,23]. Hence, evidence on the feasibility of physical activity interventions involving the whole family is very limited [24,25,26]. This important research gap needs more attention, particularly given the bidirectional relationship of physical activity behaviours between parents and children [16], and the significant influences of both parents on children’s physical activity levels [12,13,14]. 

Wearable activity trackers combined with a smartphone app have become popular for self-monitoring physical activity levels in children and adults [27,28,29]. In 2021, one in five Australian adults reported using a wearable activity tracker combined with an app (e.g., Fitbit, Garmin) [30]. These devices incorporate behaviour change techniques (e.g., goal setting, self-monitoring, real-time performance feedback, social comparison, rewards) that have been proven to influence physical activity behaviours in children and adults [31,32,33,34]. Popular activity tracker brands (e.g., Garmin Vivofit Junior and Fitbit Ace series) have also been developed age-appropriately for young children. Many recent systematic reviews and meta-analyses [31,32,33,34,35] have shown that activity trackers combined with an app effectively increase physical activity in both children and adults. Overall, this evidence suggests that activity trackers have great potential to increase physical activity in families. A key benefit of wearable activity trackers and apps is that they can be easily used by all family members to promote incidental physical activity (e.g., walking, active outdoor play) individually and within the family without the need to attend organised physical activity sessions face-to-face [22,23,25]. However, few family-based interventions have used popular commercial activity trackers and apps to promote physical activity in families [20,22,25,36]. Their results have been mixed in terms of physical activity outcomes within the family [20,22,25,36]. Therefore, the evidence base was considered inconclusive in a recent systematic review, mainly due to the small number of studies available [35].

Using popular commercial activity trackers and apps, our research team tested the feasibility of implementing an intervention (Step it Up Family) to increase physical activity in whole families [22,23]. Findings showed high (95%) family retention and significant increases in MVPA in children and both parents postintervention [23]. Further, evaluation of process data from parent surveys showed that the Garmin activity trackers and apps were popular among the children and parents [24]. This was reflected in the high ratings for usage, usability, and perceived usefulness of the activity trackers and apps [23]. Another important step towards process evaluation was to analyse the telephone interviews conducted with families postintervention to better understand their experience and satisfaction with the Step it Up Family intervention. To date, few studies [35,36] have investigated families’ experience and satisfaction with using activity trackers and apps to increase physical activity in children and parents. Particularly process evaluation using qualitative data (e.g., interviews, focus groups) is limited for technology-based physical activity interventions implemented in families [24,25]. 

Therefore, this study examined families’ experience and satisfaction with a physical activity intervention using wearable activity trackers and apps. This information, derived from qualitative intervention process data, is very useful for informing the development of future interventions using this technology to improve physical activity in children and families.

## 2. Materials and Methods

### 2.1. Study Design

This qualitative study involved semi-structured telephone interviews of parents who participated in the Step it Up Family intervention. Step it Up Family was a single-arm feasibility study with pre–post intervention measures conducted in 2017/2018 at Central Queensland University in Rockhampton, Australia [22,23]. Ethical approval for this study was granted by Central Queensland University Human Ethics Committee in May 2017 (H17/03-041). Parents provided informed online consent to participate in the Step it Up Family intervention, including this qualitative study component. 

### 2.2. Participants

Detailed information on participant recruitment is reported elsewhere [22,23]. Forty families located in Central and South-East Queensland, Australia, participated in the broader Step it Up Family study. Of these, 19 families agreed to participate in postintervention telephone interviews presented in this paper. Families were eligible to participate in the Step it Up Family study if the mother and father were aged 18 years and older, and at least one of their children was aged between 6 and 10 years. Other inclusion criteria included the following: All members of the family spoke and read English; all members of the family lived together in one household; all enrolled parents had access to the Internet as well as a smartphone or tablet; the child had not previously used an activity tracker (e.g., pedometer, Garmin, Fitbit, Apple Watch) to increase physical activity; and all members of the family could safely increase physical activity levels. Further, both parents and children had to be insufficiently active at baseline, which was defined as not meeting Australia’s physical activity guidelines (children: <60 min of MVPA per day; adults: <150 min of MVPA per week) [1,2].

### 2.3. The Step It Up Family Intervention

The Step it Up Family intervention has previously been described in detail [22,23]. Briefly, the 6-week intervention was designed to engage the whole family (mothers, fathers, children aged 6–10 years) to increase their physical activity. Key intervention components were an introductory session (60 min duration, delivered face-to-face or via telephone), wearable activity trackers and apps, and motivational and educational text messages (sent 3× per week to parents). In the introductory session and text messages, the parents and children received tips for supporting each other to increase steps and role modelling physical activity behaviours. However, primarily, Step it Up Family was an activity tracker and app intervention. Children received the Garmin Vivofit Jr tracker, including the Vivofit Jr app, and parents were given the Vivofit 3 tracker, including the Garmin Connect app. Their features have been reported in detail elsewhere [22]. Briefly, all activity trackers were waterproof, and their batteries lasted one year. The children’s activity tracker had an age-appropriate design for displaying steps and progress to reaching the recommended 60 min of physical activity a day. The corresponding children’s app (installed on parents’ smartphone or tablet) displayed the steps of all family members in a leaderboard. Additionally, the children’s activity tracker and app had other child-friendly features (e.g., bright colour band, watch, personal name and animal images on display, virtual coins as rewards, virtual adventure trail). Families were instructed to use the devices for goal setting, self-monitoring, and getting performance feedback on their daily and weekly steps and active minutes. Further, families were asked to engage in family steps challenges (individually, as a family) using the leaderboard displayed in the children’s app.

### 2.4. Data Collection

We aimed to conduct the telephone interviews in 50% of the families (i.e., one parent completed the interview on behalf of the whole family) who completed the Step it Up Family intervention. During the rolling recruitment process, we asked each family whether they would also be willing to participate in a telephone interview postintervention. A sample of 19 families (out of the 40 Step it Up Families) agreed to participate in this qualitative study component. The telephone interviews were carried out by a trained research officer (DP). The interviews were most commonly scheduled to occur within three weeks of families’ completing the intervention, at a date and time convenient to the parents. At the start of each telephone interview, it was explained to parents that all responses were valued, and that their interview responses would help improve the design of the Step it Up Family intervention. The telephone interviews lasted 15–20 min, were audio recorded, anonymised, and transcribed verbatim by a professional transcription service. A semi-structured interview questionnaire, including open-ended questions (see Supplementary Materials, was used to collect in-depth information on families’ experience and satisfaction with the Step it Up Family intervention. Questions included, for example:

“What did you like about the introductory session?”
“What did you NOT like about the introductory session?”
“What did you like about the text messages?”
“What did you NOT like about the text messages?”
“Did you experience any difficulties in using the Garmin activity trackers or Garmin apps?”
“Could you please tell us what it is about the Garmin activity trackers and apps that made you use them regularly?”
“What did you think of the features of the Garmin activity trackers and apps? What did you like about them, and what not?”

Sociodemographic data of participants were collected through online-delivered parent surveys conducted at baseline of the Step it Up Family intervention. This included child age (in years), parental age (in years), parental role (mother, father), parental education (high: 13+ years, low: 0–12 years), parental employment status (employed: full-time, part-time, casual; unemployed: home duties, student, retired), parental ethnicity (Caucasian, African, Asian; Aboriginal, Torres Strait Islanders and Pacific Islanders; Other) and location (rural/regional area, major city). In addition, data were collected on the number of children enrolled in the intervention. To provide a behavioural context surrounding the qualitative findings from the telephone interviews, data collected in the parent surveys are presented on physical activity increases postintervention within the family (in at least one parent, in at least one child, in at least one family member). Physical activity increases were defined as parents and/or children meeting the Australian national physical activity guidelines (adults: at least 150 min of MVPA per week; children aged 5–17 years: at least 60 min of MVPA per day) postintervention compared to baseline [1,2].

### 2.5. Data Analysis

Qualitative content analysis was used to identify themes, categories, and sub-categories [37,38]. Using an inductive analysis approach, an initial coding scheme was developed (by KW and SS) and informed by the semi-structured interview questions. Consistent with recommended qualitative analysis approaches [39], a researcher (KW) independent to the project delivery team initially read all the interview transcripts to obtain a sense of the whole content. Following this, the themes, categories, and sub-categories within the coding scheme were discussed and refined. Subsequently, the first researcher (KW) coded all interview data using the refined coding scheme. To insure reliability and credibility of the data analysis, a second researcher (SS) independently analysed a randomly selected 20% of the interview transcripts using the refined coding scheme. The findings from both researchers (KW, SS) were then discussed and differing data interpretations were resolved by consensus. During this process, the categories and sub-categories in the coding scheme were further refined and assigned to the raw text data [40]. Quotes were extracted from the interview transcripts to exemplify the identified categories and sub-categories. Further, to provide an indication of the prevalence of identified categories, the number of times a category was reported across all telephone interviews was presented. This approach is frequently used in qualitative content analyses [41]. Furthermore, pen profiles, which are an increasingly used technique to present analysed text data in a diagram [41,42,43], were constructed to illustrate the analysed content regarding families’ experience and satisfaction with the Step it Up Family program. Using 19 telephone interviews, thematic saturation was reached for the identified themes, categories, and sub-categories. This is consistent with empirical research [44] showing that approximately 80–92% of all concepts are identified within the first 10 interviews. Similar studies [45,46,47] using semi-structured interviews reported to have reached saturation with fewer than 20 interviews and with interview duration ranging between 10 and 40 min (in this study, interview duration was 15–20 min). The qualitative analyses were carried out in Excel (version 2201). The pen profiles were initially constructed in Word (version 2209) and then transferred into graphic design software (Adobe Illustrator, version 27.0) for better visualisation. Participants’ sociodemographic data were analysed descriptively in IBM SPSS Statistics (version 26.0).

## 3. Results

### 3.1. Sociodemographic Characteristics of Families

Table 1 presents the sociodemographic characteristics of the families (N = 19) who participated in the telephone interviews postintervention. This included 16 mothers and 3 fathers with a mean (SD) age of 38.4 years (4.8). Of the parents, 74% had 13+ years of education, 79% were employed, and 95% were Caucasian. The mean (SD) age of the children enrolled in the intervention was 7.6 years (1.4). Most families (89.5%) resided in a rural or regional area. All 19 families who participated in the telephone interviews had completed the six-week Step it Up Family intervention. Of these, 84% of families had physical activity increases (postintervention) in at least one family member, 79% of families had physical activity increases in at least one child, and 63% of families had physical activity increases in at least one parent. The sociodemographic characteristics of this sub-sample of 19 families (Table 1) were similar to the sociodemographic characteristics of all 40 families participating in the intervention [22].

### 3.2. Experience with the Activity Trackers and Apps

**Figure 1:**
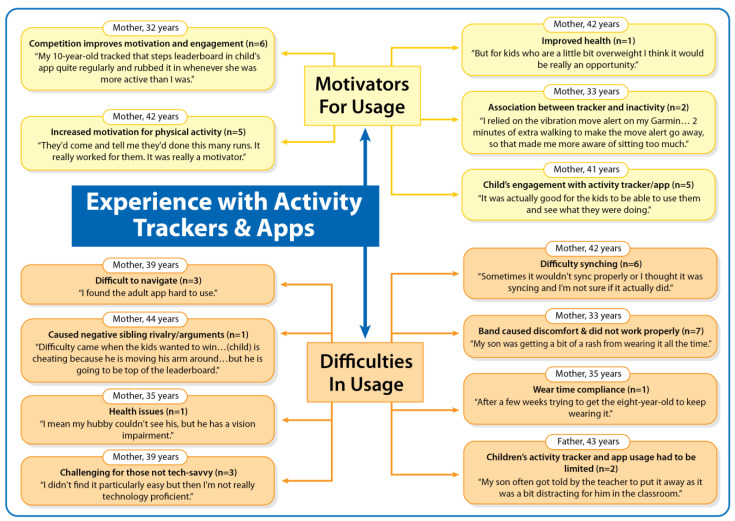
Families’ experience with the activity trackers and apps.

Families’ experience with the activity trackers and apps is illustrated in Figure 1. Parents reported on their family’s experience, which was expressed predominantly in terms of motivators and difficulties in using the activity trackers and apps.

#### 3.2.1. Motivators for Usage

Parents (n = 6) reported that the competition during the family step challenges increased their motivation and engagement with the activity trackers and apps.

*My 10-year-old tracked that steps leaderboard in child’s app quite regularly and rubbed it in whenever she was more active than I was*.

[Mother, 32 years; physical activity increased within the family]

Parents (n = 5) positively noted that the children, in particular, engaged with the activity tracker and app features to reach their daily step goals.

*It was actually good for the kids to be able to use them and see what they were doing*.

[Mother, 41 years; physical activity increased within the family]

Parents (n = 5) also reported that the activity trackers and apps increased physical activity motivation among the children and parents.

*They’d come and tell me they’d done this many runs. It really worked for them. It was really a motivator*.

[Mother, 42 years; physical activity increased within the family]

Some parents (n = 2) found that the association between activity tracker alerts and physical inactivity was a motivation to start moving.

*I relied on the vibration move alert on my Garmin…2 min of extra walking to make the move alert go away, so that made me more aware of sitting too much*.

[Mother, 33 years; physical activity increased within the family]

#### 3.2.2. Difficulties in Usage

Several parents (n = 7) reported that the activity tracker band caused discomfort and/or sometimes did not work properly.

*My son was getting a bit of a rash from wearing it all the time… it would come undone really easily*.

[Mother, 33 years; physical activity increased within the family]

*The child’s band broke part way through.*

[Mother, 44 years; physical activity increased within the family]

Parents (n = 6) had difficulties with syncing the recorded activity tracker data to the apps.

*Sometimes it wouldn’t sync properly or I thought it was syncing and I’m not sure if it actually did*.

[Mother, 42 years; physical activity increased within the family]

Some parents (n = 3) found it difficult to navigates the apps.

*I found the adult app hard to use.*

[Mother, 39 years; physical activity did not increase within the family]

Some parents (n = 3) found the activity tracker and app use challenging for those who are not tech-savvy.

*I didn’t find it particularly easy but then I’m not really technology proficient*.

[Mother, 39 years; physical activity did not increase within the family]

Some parents (n = 2) reported that the children’s activity tracker and app use had to be limited when it became distracting or too excessive.

*My son often got told by the teacher to put it away as it was a bit distracting for him in the classroom.*

[Father, 43 years; physical activity increased within the family]

One parent reported difficulties with their child’s compliance to wear the activity tracker.

*After a few weeks trying to get the eight-year-old to keep wearing it.* [Mother, 35 years; physical activity did not increase within the family]

Another parent noted that the step counting caused negative sibling rivalry and arguments.

*Difficulty came when the kids wanted to win…(child) is cheating because he is moving his arm around…but he is going to be top of the leaderboard*.

[Mother, 44 years; physical activity increased within the family]

### 3.3. Satisfaction with the Step it Up Family Intervention Components

**Figure 2:**
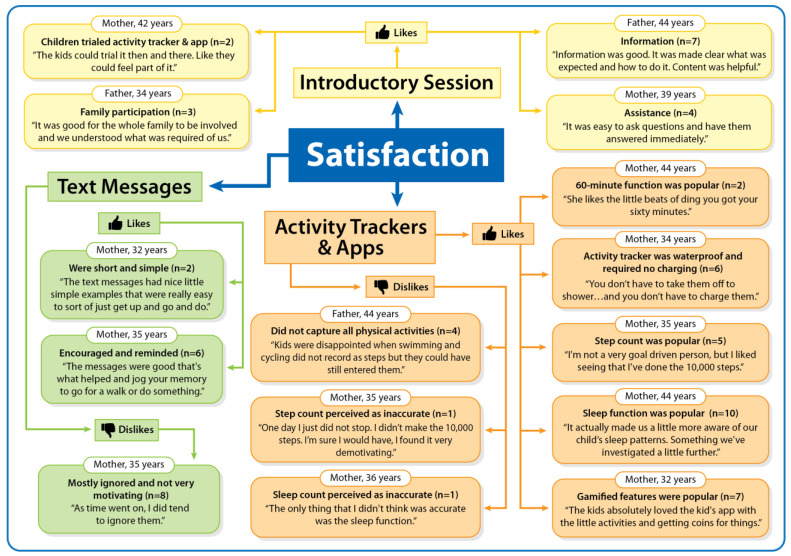
Families’ satisfaction with the Step it Up Family intervention components.

Families’ satisfaction with the Step it Up Family intervention components is presented in Figure 2. Parents reported on their family’s (dis)satisfaction regarding key intervention components: the introductory session, activity trackers, apps, and text messages.

#### 3.3.1. Introductory Session

Parents (n = 7) liked the information provided in the introductory session.

*Information was good. It was made clear what was expected and how to do it. Content was helpful*.

[Father, 44 years; physical activity increased within the family]

Furthermore, parents (n = 4) liked the practical assistance provided during the introductory session and the opportunity to ask questions.

*It was easy to ask questions and have them answered immediately.*

[Mother, 39 years; physical activity did not increase within the family]

Further, parents (n = 3) liked that the introductory session provided an opportunity for all family members to be involved.

*It was good for the whole family to be involved and we understood what was required of us*.

[Father, 34 years; physical activity increased within the family]

Some parents (n = 2) liked that the children could trial the activity tracker and app during the introductory session.

*The kids could trial it then and there. Like they could feel part of it*.

[Mother, 42 years; physical activity increased within the family]

#### 3.3.2. Activity Trackers and Apps

Parents (n = 6) repeatedly noted that they liked that the activity trackers were waterproof and required no charging.

*You don’t have to take them off to shower…and you don’t have to charge them*.

[Mother, 34 years; physical activity did not increase within the family]

Parents (n = 5) reported that the step count displayed in activity trackers and apps were a popular feature among the children and parents.

*I’m not a very goal driven person, but I liked seeing that I’ve done the 10,000 steps*.

[Mother, 35 years; physical activity increased within the family]

Many parents (n = 10) liked that the activity tracker and apps also monitored sleep duration and patterns (e.g., falling asleep, wakefulness at night) of both the children and parents.

*It actually made us a little more aware of our child’s sleep patterns. Something we’ve investigated a little further*.

[Mother, 44 years; physical activity increased within the family]

Several parents (n = 7) noted that their children liked the gamified features (e.g., getting coins, virtual adventure trail) in the child’s activity tracker and app.

*The kids absolutely loved the kid’s app with the little activities and getting coins for things.*

[Mother, 32 years; physical activity increased within the family]

Some parents (n = 2) noted that their children liked the 60 min function (i.e., a circle displayed when 60 active minutes were achieved) in the child’s activity tracker.

*She likes the little beats of ding you got your sixty minutes.*

[Mother, 44 years; physical activity increased within the family]

In terms of dislikes, some parents (n = 4) reported their children disliked that the activity trackers and apps did not capture all physical activities, such as swimming and cycling.

*Kids were disappointed when swimming and cycling did not record as steps but they could have still entered them*.

[Father, 44 years; physical activity increased within the family]

#### 3.3.3. Text Messages

Parents (n = 6) liked that the text messages encouraged and reminded them to be active.

*The messages were good that’s what helped and jog your memory to go for a walk or do something*.

[Mother, 32 years; physical activity increased within the family]

Further, some parents (n = 2) liked that the text messages were short and simple.

*The text messages had nice little simple examples that were really easy to sort of just get up and go and do*.

[Mother, 32 years; physical activity increased within the family]

However, many parents (n = 8) reported that they did not find the text messages very motivating, and so they mostly ignored the text messages.

*In regard to the text messages, they were neutral on the benefits of those. For them it wasn’t motivating*.

[Mother, 44 years; physical activity increased within the family]

*I did read them, but probably didn’t implement them, I guess.*

[Mother, 41 years; physical activity increased within the family]

## 4. Discussion

This qualitative study examined families’ experience and satisfaction with a family-based physical activity intervention using activity trackers and apps. In terms of experience, parents reported that the activity tracker and apps increased their family’s physical activity motivation. This was further enhanced by the competitive components in the intervention (i.e., family steps challenges), which promoted family members’ engagement with the activity tracker and apps. The children, in particular, engaged with various activity tracker and app features (e.g., step count, 60 min function, family leaderboard) to reach their daily step goals. This qualitative finding reported by parents in the telephone interviews corresponds to the quantitative findings from objective activity tracker data published previously on this study, which showed that children’s and parents’ activity tracker and app usage was high throughout the 42-day intervention period (i.e., step recordings were, on average, 37 days in children and 38 days in parents) [22]. Some technical difficulties were reported, for example, when navigating the apps, syncing the recorded activity data to the apps, and when the activity tracker band caused discomfort or did not work properly. In terms of satisfaction, families liked several activity tracker functions (i.e., step count, sleep monitoring, being waterproof, no charging required) and gamified features in the children’s app (e.g., getting coins, virtual adventure trail). However, they disliked that the activity tracker did not capture all physical activities (e.g., swimming, cycling). Further, parents liked that the text messages reminded them to be active, but they did not find them motivating, and so, they often ignored the text messages.

Steps competitions between children and parents increased families’ physical activity motivation. In particular, the gamified features in the child app (e.g., getting coins, virtual adventure trail, family leaderboard) were well-received by the children, and this promoted their engagement with the activity tracker and app. This aligns with many technology-based physical activity interventions’ [42,48,49,50] showing that social comparison and gamification embedded in activity trackers and apps enhances participants’ intervention engagement. In turn, high intervention engagement is known to positively influence interventions’ effects on physical activity [51]. As such, it may be that children’s positive experience and satisfaction with the activity tracker and app contributed to their engagement with the devices, which in turn may have contributed to the significant physical activity increases detected postintervention [22]. Another activity-tracker-based intervention [24] has also shown that friendly intra-family competitions (e.g., for steps) motivate family members’ physical activity participation. There is potentially a concern for intra-family steps comparisons to become too competitive and thereby create sibling rivalry rather than a friendly competition. However, this problem was expressed by only one family in this study. A solution may be that parents moderate the family steps challenges and interfere when sibling competitions become too fierce.

The user-friendly functions (i.e., being waterproof, no recharging required) of the activity trackers were well-received by families. However, technical difficulties (i.e., with navigating the app, syncing the recorded activity data to the apps, an uncomfortable or dysfunctional tracker band) were also noted, which may impede engagement with the devices in some families. Users’ desire for waterproof activity trackers with lasting battery life has been reported in other studies [36,41,42], as was difficulties with syncing activity tracker data to a corresponding app and problems with broken tracker bands [36,41,42]. These findings highlight the importance of functionality and wearability of activity trackers, particularly among children, who will likely forget to recharge an activity tracker or take it off during water-based activities.

Families varied in their satisfaction with the step count function of the activity tracker. Families liked the step counting, but they disliked that it did not capture all physical activities (e.g., cycling, swimming). This finding is consistent with those reported in other family-based activity tracker interventions [36,41]. The fact that step counting does not capture all physical activities children often engage in may impede the usage of activity trackers in some families [41,42], and this may result in underestimation of physical activity levels [36]. This is a common limitation of wearable activity trackers. It could be addressed, though, by providing users the opportunity to log active minutes accumulated through cycling and water-based activities manually in an app. The app could then automatically convert the active minutes to steps. This approach is currently being applied in the Australian 10,000 Steps program, which is a large-scale community-based program aiming to promote physical activity by counting steps through a website (10000steps.org.au (accessed on 1 January 2023)), app and activity trackers [52].

Families liked that the text messages reminded them to be active, but they did not find them very motivating, and therefore, often ignored the text messages. That families like to receive text messages as a reminder to be physically active was also found in another family-based physical activity intervention using text messages [53]. Generally, text-message-based interventions have shown to positively influence physical activity behaviour in children and adolescents [54]. Hence, text messages may still be a viable intervention strategy, also because parents are interested in receiving text messages with health information for their children [55,56]. However, the content of the text messages in the Step it Up Family intervention needs improvement and further testing in families. Perhaps, it is better to co-create text messages with children and families [55], include pictures, videos, or links to more information in the text messages [56], and allow families to decide when and how many text messages they would like to receive. Another option is to provide personalised text messages tailored to family members’ physical activity progress throughout the intervention [55]. This may better integrate the technology-based intervention components of ‘text messages’ and ‘activity trackers and apps’. Another possible way of motivating families to increase their physical activity may be to interact more dynamically with them through dialogue (e.g., via an artificially intelligent virtual assistant). With technological advances in machine learning and artificial intelligence, digital communication with participants through an artificially intelligent virtual health assistant (i.e., chatbot) has shown promise to support people to become more physically active [57,58].

An additional finding in this study was that many parents liked the sleep function (i.e., monitoring sleep duration and patterns) in the activity tracker and apps. This is understandably important to parents, given that sleep deprivation and sleep disruptions are a major health issue among children and parents [59]. Parents’ interest in sleep monitoring with an activity tracker has also been noted in similar studies [41,42] that utilised activity trackers to promote physical activity in children and adolescents. Future family-based activity tracker and app interventions may consider the use of wearable activity trackers to improve sleep behaviours in the family context.

A strength of this qualitative study was the in-depth insights into families’ experiences and satisfaction with using wearable activity trackers and apps to improve physical activity levels within the family. Few studies [25,35,36] have explored experiences and satisfaction with using this technology to promote physical activity in families. This study also has several limitations. Firstly, the qualitative data were obtained from a sample of families that were mostly highly educated, employed, and Caucasian. Therefore, the findings cannot be generalised to the context of all family populations. Secondly, all families used the same Garmin Vivofit activity trackers and app (i.e., model version Vivofit 3 for parents, Vivofit Jr for children). Hence, the satisfaction and experience reported by parents is limited to this specific activity tracker and app brand and model version. Thirdly, the satisfaction and experiences with using activity trackers and apps was limited to families with children aged 6–10 years. Families with adolescents may have expressed different experiences and satisfaction with using activity trackers and apps to become physically active. Future studies may test the feasibility, acceptability, experience, and satisfaction with using activity trackers and apps in families with children across various age groups (preschool, primary school, secondary school children). Using an adequately powered sample size, future studies may also investigate whether families’ experiences and satisfaction with an activity tracker and app-based intervention align with behavioural outcomes (i.e., increases in physical activity levels). This is worth exploring, as what families like/dislike in terms of activity tracker and app features and what effectively increases their physical activity may differ. Furthermore, we recommend that future activity tracker and app-based interventions to increase physical activity in families embed gamification components (e.g., family step challenges, rewards for achieving step goals) to promote intervention engagement. To investigate intervention effectiveness, future studies should be conducted in larger samples of families using a rigorous randomised controlled trial design, objective physical activity measurement (e.g., by accelerometry) and a long-term intervention period with multiple follow-ups.

## 5. Conclusions

Families’ experience and satisfaction with the Step it Up Family intervention was generally positive. The use of wearable activity trackers and their corresponding apps increased physical activity motivation among both children and parents. This was further enhanced through the friendly competitions (i.e., family step challenges) which promoted family members’ engagement with the activity trackers and apps. The children, in particular, engaged with various activity tracker and app features (e.g., step count, 60 min function, family leaderboard) to reach their daily step goals. The user-friendly functions (i.e., being waterproof, no recharging required) of the activity trackers, as well as the gamified features in the children’s app (i.e., family leaderboard, getting coins, virtual adventure trail) appeared to have played a crucial role in families’ positive experience and satisfaction with the devices. However, technical difficulties with navigating the app, syncing the activity tracker data to the app, and a dysfunctional tracker band may have undermined families’ positive experience, which shows the importance of user-friendly and functional designs for positive user experiences. Further, the inability of activity trackers to capture cycling and water-based activities children often engage in somewhat impedes families’ satisfaction with these devices. The weekly text messages sent throughout the intervention were reported to be useful reminders to be physically active. However, they failed to be motivating and therefore need improvement and further testing in families. Overall, the findings from this study can inform further refinement of interventions to improve physical activity in families at a larger scale using technology-based approaches (i.e., activity trackers, smartphone apps, websites).

## Figures and Tables

**Table 1:** Sociodemographic characteristics of families.

N (Families)		19
Parental age, M (SD)		38.4 (4.8)
**Child age, M (SD)**		7.6 (1.4)
**Parental role, n (%)**		
	Mother	16 (84.2)
	Father	3 (15.8)
**Parental education, n (%)**		
	13+ years	14 (73.7)
	0–12 years	5 (26.3)
**Parental employment status, n** **(%)**		
	Employed	15 (78.9)
	Unemployed	4 (21.1)
**Parental ethnicity, n** **(%)**		
	Caucasian	18 (94.7)
	Asian	1 (5.3)
**Location, n (%)**		
	Rural/Regional area	17 (89.5)
	Major city	2 (10.5)
**Number of children enrolled in the intervention, n (%)**		
	One child	10 (52.6)
	Two children	9 (47.4)
**Physical activity increases postintervention, n (%)**		
	In at least one parent within the family	12 (63.16)
	In at least one child within the family	15 (78.9)
	In at least one member within the family	16 (84.2)

Abbreviations: M = mean, SD = standard deviation.

## Data Availability

The data presented in this study may be available upon reasonable request from the corresponding author.

## Supplementary Materials

The following supporting information can be downloaded at https://www.mdpi.com/article/10.3390/ijerph20043327/s1. File S1: Semi-structured telephone interviews—procedure, introduction, and questions.
