# Supplementary material for: Experience and Satisfaction with a Family-Based Physical Activity Intervention Using Activity Trackers and Apps: A Qualitative Study
Source: Int J Environ Res Public Health. 2023 Feb 14;20(4):3327. doi: 10.3390/ijerph20043327 (PMC9963519; doi:10.3390/ijerph20043327)
Supplement: Supplementary file 1 [file ijerph-20-03327-s001.zip › ijerph-2128946-supplementary.pdf]

Supplementary Materials

Semi-structured telephone interviews - procedure, introduction and questions

Note, all questions asked in the telephone interviews are presented in the following for completeness. However, only text data in relation to questions relevant to families' experience and satisfaction with the Step it Up Family program was analysed and presented in his paper.

Procedure

Short (15-20 minutes) semi-structured telephone interviews will be conducted with at least 15 parents and 15 children from both families that demonstrated good improvements in physical activity as well as from families that demonstrated little improvement in physical activity. The aim is to assess what worked and what not and how future physical activity interventions using activity trackers and apps should be developed to optimally to suit families.

Introduction

(Put this in your own words, needs to sound natural to you)

Hello <name participant>,

This is <Stephanie>, from the Step it Up Family Program! I would like to ask you a couple of questions about your participation in the Step it Up Family Program. Don't worry, it won't take long, 15 to 20 minutes maximum! Your participation will help us a lot in understanding the outcomes of the study; it is about what you liked and didn't like about the program. Are you happy to help us out right now or do you want to do it at a later time?

If no: when would be a better time; later today perhaps?

If yes: excellent! Let's get started!

If they refuse to participate: Ask why, and also if that is the same reason why were not or little participating in the program.

Before we start I would like to point out that there are no good or bad answers to any of the questions I will ask. Please be really honest in your answers even if you think it is not what we want to hear, all feedback is valuable! OK here we go:

1. What did you like about the face-to-face information session?
2. What did you NOT like about the face-to-face information session?
3. What did you like about the text messages?
4. What did you NOT like about the text messages?
5. Were the text messages sent often enough?

6. Was the time of the day the text messages were sent appropriate?
7. Did you experience any difficulties in using the Garmin activity trackers or Garmin apps? If so, what were they?
8. Could you please tell us what it is about the Garmin activity trackers and apps that made you use them regularly?
   Prompt: Make sure they say something about the Garmin activity trackers and Garmin apps!
9. What did you think of the features of the Garmin activity trackers and apps? What did you like about them, and what not?
   Prompt: Make sure they say something about what they liked AND not liked!
10. What type of physical activities did you, the mother/father, and your child/ren do to increase your steps?
11. Were all family members trying to increase their steps whilst using the Garmin activity tracker and app?
    Prompt: Make sure they say something about mother's, father's and child/ren's efforts to increase steps.
12. Were you, the mother/father, able to support your child to increase his/her steps?
    If yes
    How did you do it?
    If no
    What prevented you from supporting your child to increase his/her steps?
13. Were you, the mother/father, able to model active behaviours to your child in order to increase his/her steps?
    If yes
    How did you do it?
    If no
    What prevented you from modelling active behaviours to your child in order to increase his/her steps?
14. How do you think the program has influenced the your families' physical activity levels?
    Prompt: Specifically ask about the physical activity levels of the mother, father and child/ren.
15. Which aspects of the program did you find helpful to increase your physical activity?
16. What did you expect to get out of the program?
17. Did the program meet your expectations?
18. What would you suggest to improve the program?
19. Did you encounter any difficulties with the program? If so, what were they?
20. Do you have any other comments, suggestions or feedback that would improve the program?

OK, that is all I wanted to ask! Thank you for your cooperation!
